# Clinical preceptorship in Ghana in the era of COVID-19 pandemic: an interventional study

**DOI:** 10.1186/s12912-022-00838-w

**Published:** 2022-03-14

**Authors:** Nancy Innocentia Ebu Enyan, Christian Makafui Boso, Anastasia Adomah Ofori, Dorcas Frempomaa Agyare, Irene Korkoi Aboh, Nkechi Oluwakemi Dike, Douglas Darko Agyei, Dorcas Obiri-Yeboah

**Affiliations:** 1 Department of Adult Health, School of Nursing and Midwifery, University of Cape Coast, Cape Coast, Ghana; 2 Department of Medical Education and I.T, School of Medical Sciences, University of Cape Coast, Cape Coast, Ghana; 3 Department of Mathematics and ICT Education, Faculty of Science and Technology Education, University of Cape Coast, Cape Coast, Ghana; 4 Department of Microbiology, School of Medical Sciences, University of Cape Coast, Cape Coast, Ghana; 5 Directorate of Research, Innovation and Consultancy, University of Cape Coast, Cape Coast, Ghana

**Keywords:** Coronavirus pandemic, Developing countries, Intervention, Preceptorship, Technology

## Abstract

**Background:**

Innovations in clinical nursing education are critical in enhancing the experiences of students, especially in the era of coronavirus pandemic. This study aimed at investigating nurse preceptors’ perceptions of use, intention to use and self-efficacy towards digital technology in preceptorship in the Cape Coast Metropolis of Ghana.

**Methods:**

A concurrent type of mixed-methods design with a non-randomised interventional study using three-phase multi-methods technique was conducted among nurse preceptors in the Cape Coast Metropolis in the Central Region of Ghana. Forty-five nurse preceptors participated in a pre and post training intervention while seven were purposively selected for the qualitative interview. Complimentarity method of triangulation was used in the analysis. The quantitative data were analysed with STATA version 16 and presented using frequencies, percentages, means with standard deviations and McNemar's test while qualitative data were analysed using the six steps approach to qualitative data analysis by Braun and Clarke.

**Results:**

Perceived usefulness statement “using technology will improve clinical teaching” increased from 19 (42.22%) at baseline to 44 (97.78%) post intervention. Perceived ease of using technology statement “I would find it easy to get this technology to do what I want it to do” also increased from 36 (80.00%) to 41 (91.11%) post intervention. Self-efficacy increased from 40 (88.89%) to 43 (95.56%) after the intervention. There was a statistically significant difference between pre-training and post-training scores regarding tablet (*p* = 0.016) and experience with online library resources (*p* = 0.039). The qualitative results yielded three themes, namely: strengths of using technology; constraints in the learning environment; and future of technological approach to clinical teaching.

**Conclusions:**

The training intervention improved participants intentions, self-efficacy, perceived use and perceived ease of use of technology. However, there are constraints in the clinical learning environment including students and preceptor-related factors, and institutional factors that needs to be addressed as part of efforts to implement technology in clinical teaching in this era of COVID-19 pandemic and beyond.

## Background

Nursing education programmes rely heavily on high quality clinical preceptors to provide trainees with real life clinical experiences, and to guide them to growing professionally into their eventual roles as part of the healthcare team. Preceptorship is critical to nursing and health sciences education, especially in high-income economies where it an established concept [1]. Clinical preceptorship involves a developmental relationship between an experienced and knowledgeable individual (the preceptor) and a trainee (the preceptee), whereby the trainee acquires clinical competencies through constructive guidance and support [2]. A preceptor in this paper refers to an experienced nurse or midwife who supports students learning at the clinical area through teaching and supervision thereby bridging the gap between theory and practice. The preceptor closely works with the preceptee who is a student with the need to acquire specific clinical competencies in order to achieve a set of educational goals regarding their clinical training [2].

The transition process, which also has a socialization component, occurs through observation and role-modeling behavior within the learning environment. Students participate in teamwork, decision-making, assessments, problem solving, critical thinking, coping with actual patients and their problems, and applying theoretical knowledge in actual practice [3].

Despite preceptors’ vital role in nursing education, they have their unique set of challenges in carrying out their responsibilities in the clinical environment, especially in settings like Africa. Preceptors are burdened with heavy workload, have no or limited access to refresher preceptor courses and contemporary concepts of learning using technology. This leads to a lack of active participation by learners, and less time assessing and assisting students on the ward [4–6].

In Ghana, preceptorship has not been well implemented partly due to lack of equipment for clinical teaching and learning and inadequate support for the preceptor role [7]. Nonetheless, a study conducted in the Cape Coast Metropolis of Ghana reported that majority of the nurses and midwives had the intention of functioning in the preceptor role in the near future, yet they had not been trained to effectively perform the role [8].

Additionally, the coronavirus disease (COVID-19), in light of weak preceptorship in health sciences education in Ghana, presents a special challenge to clinical education that requires concerted efforts to rethink and modify the conventional approaches to meet this challenge. Before the COVID-19 pandemic, major challenges confronting clinical education included large students’ numbers at the clinical settings and limited numbers of trained preceptors [9, 10].

Effective intervention to the global COVID-19 pandemic in the area of nursing education is digitalization which requires the application of digital technology in transforming existing services [11]. With the challenge posed by the COVID-19 pandemic, it has become necessary that digitalisation and virtual learning platforms are adopted to bring the needed transformation and improvement [12]. Training of nurse preceptors on the use of digital technology will augment the efforts of the academic faculty, as well as adapt to the changing trends the pandemic imposes, through increasing use of digitalization of the roles previously played in direct physical contact. This study, therefore, sought to investigate nurse preceptors’ 1) ownership and experience with the use of digital technologies, 2) perceptions, intention to use and self-efficacy towards digital technology in preceptorship and 3) challenges to the use of technology in preceptorship in the Cape Coast Metropolis of Ghana.

## Methods

### Study design and setting

This study adopted the concurrent type of mixed-methods design with a non-randomised interventional study using three-phase multi-methods technique. Phase one covered baseline data of the participants. Phase two focused on the training intervention and phase three covered the post intervention quantitative data collection and qualitative interviews. The interval between baseline data collection and posttest was six weeks. Three months after the posttest, a descriptive qualitative study was conducted as part of the phase three to explore the views of nurse preceptors regarding their perceptions and challenges to the use of technology in preceptorship.

This study was conducted in the Cape Coast Metropolis which is located in the southern part of Ghana. The Metropolis has three educational institutions that provides nursing and midwifery education. Students from these training institutions receive clinical training and mentoring from experienced clinicians and preceptors in the clinical placement sites within the Metropolis.

### Study population

The population comprised practicing nurses and midwives, or clinicians with interest in clinical training of nursing and midwifery students in the Cape Coast Metropolis. The eligibility criteria were nurses and midwives with a minimum of three years’ experience who have been engaged as nurse preceptors; and those who have shown interest and demonstrated the desire to teach nursing and midwifery students while on clinical placement in the Cape Coast Metropolis. Overall, 45 nurse preceptors participated in the quantitative aspect of the study while seven participated in the qualitative study.

The study covered the following socio-demographic information; gender, age, duration of practicing nursing /midwifery, duration of being involved in clinical teaching, type of clinical teaching site, clinical specialty and rank.

### Sample size and sampling

Sampling for this study was conducted in phases. For the quantitative phase of the study, all 51 who met the inclusion criteria were invited. However, 45 consented and therefore participated in the study. The nurse managers in the clinical facilities assisted the research team in selecting the participants for the study using the inclusion criteria. The qualitative phase of the study involved all the nurse preceptors from the different health facilities who were assigned students to teach via technology during the intervention phase of the project. Data saturation was achieved after interviewing the fifth participant; however, two more participants were interviewed to confirm data saturation. At the end, seven nurse preceptors participated in the qualitative study.

### Data collection instruments

The instrument used for this study was adapted from the technology acceptance model questionnaire developed by Davis in 1989 [13]. Literature was reviewed and additional items on technology and teaching added by the research team [14, 15]. The instrument covered questions on types of digital technologies used by preceptors, as well as the digital devices owned by preceptors. The perceived usefulness of technology in clinical teaching subscale comprised 6 items on a seven-point Likert scale, ranging from strongly agree to strongly disagree. Perceived ease of use of technology in clinical teaching comprised 4 items on a seven-point Likert scale, ranging from strongly agree to strongly disagree. Intention to use technology in clinical teaching consisted of 2 items and self-efficacy was measured with a single item all on a seven-point Likert scale. The instrument was pretested on 10 nurses in a nearby hospital. The few questions that the participants had difficulty answering were reworded before using it for the main study. Face validity of the instrument was achieved by ensuring that the items on the instrument reflect the objectives of the study. The instrument had a reliability coefficient of 0.901 in the present study.

A semi-structured interview guide was developed for the qualitative interviews for the nurse preceptors. The quantitative study inspired the development of this guide as the qualitative interviews aimed at exploring participants’ perceptions of using technology for clinical teaching. The guide covered the following broad questions: a) Can you tell me your perception of using technology in teaching students on the ward? b) How has the use of technology influenced your task achievement during clinical teaching sessions? c) Can you tell me about the challenges you encountered incorporating technology in your clinical teaching sessions? d) Describe some instances you enjoyed most during the use of technology in your teaching sessions? e) Generally, what is your view about the continuous use of technology during clinical teaching of students? The guide was pretested on two nurses and necessary amendments made before the actual data collection.

## Data collection procedure

### Phase one: pre-training baseline data

Phase one of this study involved the collection of baseline data on nurse preceptors’ perceptions of use, intention to use and self-efficacy towards digital technology in preceptorship in the Cape Coast Metropolis of Ghana using a questionnaire. The baseline data informed the content of the training.

### Phase two: intervention

Phase two of the multi-methods approach involved a two-day training, both face to face and virtual on the use of technology in clinical teaching, the concept of preceptorship, the roles of preceptors and preceptees, the motivators and the challenges of being a preceptor. The session of the training on technology and clinical teaching lasted for two hours while the second session on preceptorship lasted for approximately four hours. After the training intervention, eight nurse preceptors were assigned a group of eight to ten level 200 students from a public university that runs a bachelor of science in nursing programme. These selected participants were tasked to precept the students using the knowledge gained on clinical teaching via technology and preceptorship. The clinical course guide for the level 200 s were shared and discussed with the participants to have a better understanding of the objectives and expectations of the students. The nurse preceptors engaged with the students three times a week, for a maximum of two hours per session. These sessions scheduled after each clinical session, involved the use of technology, and spanned a total of six weeks. The participants were supplied with internet data. The nurse preceptors used their own devices including smartphones, tablets, laptops, etc. Additionally, one culturally appropriate video on taking up and handing over a ward was developed as part of the intervention. The preceptors were to use it in clinical teaching.

### Phase three: post intervention

**Fig. 1:**
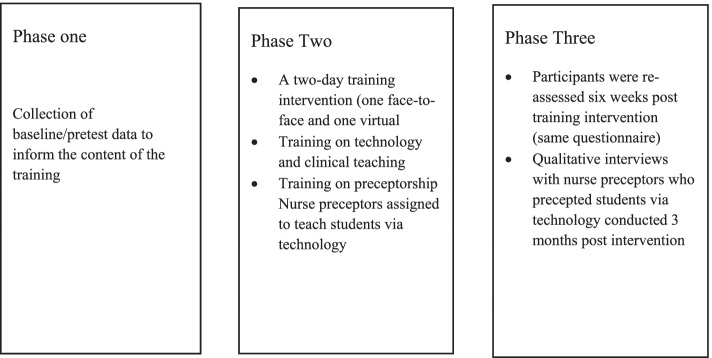
A summary of the phases of the project

In phase three, the participants were reassessed six weeks after the training intervention using the same questionnaire. Additionally, qualitative interviews were conducted three months post intervention. This involved face-to-face interviews that took place at a convenient place, date and time agreed upon by the preceptors and researchers. The interviews were audio-recorded with permission from the participants. In all, seven nurse preceptors participated in the study. Field notes were taken to augment the data from the interviews. Each interview session lasted approximately 20 to 60 min. The interviews were conducted in the English language. Figure 1 provides a summary of the phases of the project.

## Data Analysis

Complimentarity method of triangulation was used in the analysis to develop a richer understanding of the results by allowing the results of both the quantitative and qualitative aspects of the study to inform each other [16]. The questionnaires were checked for completeness by two persons. The data were entered and cleaned to correct any discrepancies before proceeding with the analysis. The data were analysed with STATA version 16 and presented using frequencies and percentages. Based on normality, means with standard deviations were presented where indicated and McNemar's test was used where appropriate and the *p*-value presented.

**Fig. 2:**
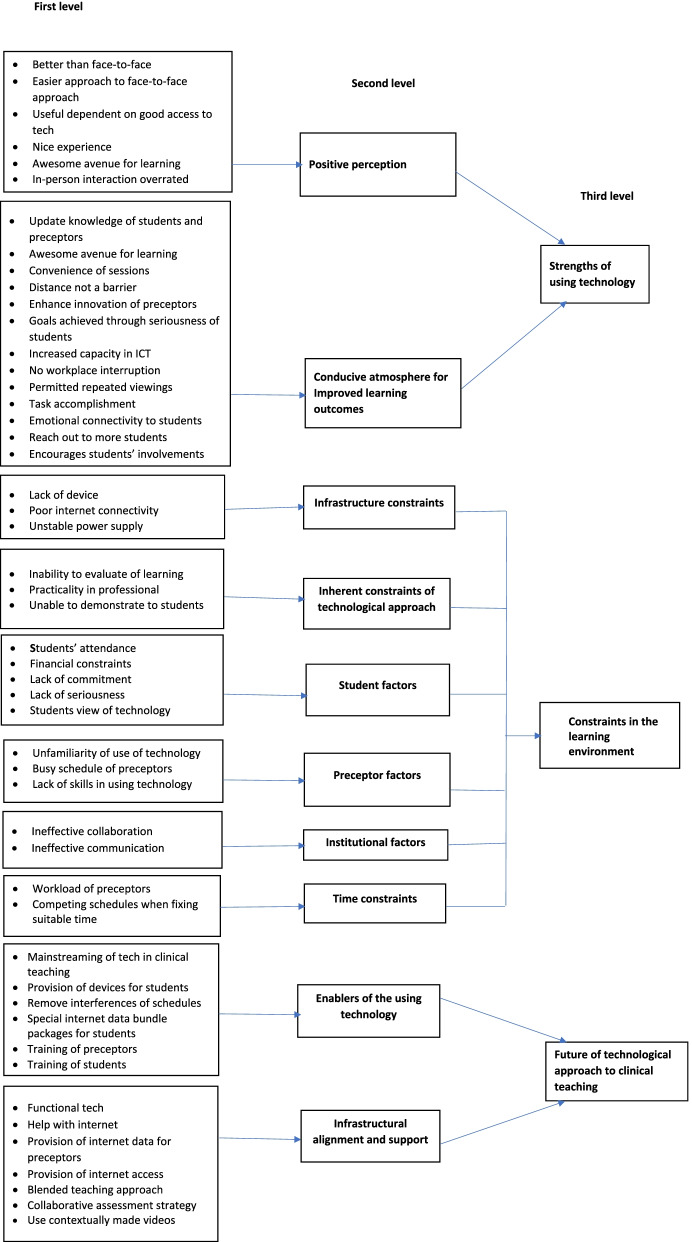
The coding tree

Using Braun and Clarke’s six steps of qualitative data analysis which involves reading and familiarization with the data, coding, searching for themes, reviewing the themes, defining and naming the themes and finalizing the analysis [17], the second and third authors separately undertook a manual data analysis. The second author transcribed verbatim the audio-recorded interviews after which all participants were requested to cross-check the transcripts for accuracy as recommended by Tong, Sainsbury, and Craig [18]. Step one of Braun and Clarke’s analysis deals familiarization of oneself with data. Here, the two authors simultaneously listened to the audio and read the transcripts to familiarize themselves with the data. They carried additional three reads of the transcripts. The second step included generating initial codes. The second and third authors assigned codes by highlighting sentences or phrase that related to the objectives of the study. Thirdly, the codes were organized into the main points recurring throughout the data. The main points were then categorized into themes and subthemes. During the fourth step, themes and subthemes were checked with the coded extracts. Also, fine tuning of the themes and subtheme were done. Furthermore, a cross-checking of the themes and subthemes with the transcripts were carried out by the second and third authors for accuracy. During the fifth step, theme and subtheme names and definitions that reflected the crux of the themes and subthemes were provided. The final step involved the extraction of indicative statements from the transcripts to support the themes. Figure 2 presents the coding tree.

A number of measures were put in place to ensure the trustworthiness of the study. First, the interview guide was pre-tested with preceptors in analogous facilities to fine-tuning of the questions. Through the pre-testing prompts were included to questions to encourage participants to address all issues relating to the study. Also, the interviewer-built rapport with the participants through several interactions before interviews were conducted. Two blinded data analysis and participant checking were carried out consistent with Tong et al.’s recommendation [18]. Indicative statements were used to support themes and subthemes. Additionally, the second and third authors diligently scrutinized the coding and themes in relations to the transcripts.

## Results

In all, 45 participants were involved in the pretest–posttest and the intervention. Table 1 shows the socio-demographic and other relevant characteristics of the participants for the quantitative aspect of the study. The majority of the participants were within the age range 31 to 40 years (*n* = 28,62.22%). The mean age of the participants was 39.48 with a standard deviation of 6.05. Regarding work experience as practicing nurse, 33 (73.33%) have worked for 11 to 20 years and most of them 20 (44.44%) had clinical nursing teaching experience of between 6 to 10 years. Additionally, 20 (44.44%) were principal nursing officers with only few 4(8.89%) being nursing officers.

**Table 1:** Socio-demographic and other relevant characteristics of study participants (*N* = 45)

Variable	Frequency (Percentage) *n* (%)
**Age (years)**	
Mean (SD)	39.48 (6.05)
≤ 30	2 (4.44)
31–40	28 (62.22)
41–49	12 (26.67)
≥ 50	3 (6.67)
**Gender**	
Female	38 (84.44)
Male	7 (15.56)
**Work experience as a practicing nurse (years)**	
Mean (SD)	14.53 (5.89)
≤ 10	8 (17.78)
11–20	33 (73.33)
21–30	4 (8.89)
**Nursing clinical teaching experience (years)**	
Mean (SD)	8.09 (4.17)
≤ 5	15 (33.33)
6–10	20 (44.44)
11–15	9 (20.00)
≥ 16	1 (2.22)
**Nursing Rank**	
Nursing Officer	4 (8.89)
Senior Nursing Officer	16 (35.56)
Principal Nursing Officer	20 (44.44)
Deputy Director of Nursing Services	5 (11.11)
**Level of Practicing facility**	
District Hospital	17 (37.78)
Teaching Hospital	28 (62.22)
**Nursing Specialty**	
Critical Care/Emergency	10 (22.22)
EENT	4 (8.89)
General Nursing	18 (40.00)
Midwifery	5 (11.11)
Perioperative Nursing	4 (8.89)
Psychiatry	1 (2.22)
Public Health	3 (6.67)

Table 2 shows a statistically significant difference between pre-training and post-training scores regarding tablet (*p* = 0.016) and experience with online library resources (*p* = 0.039). However, there was no statistically significant difference between the pretest and posttest scores for other commonly owned digital devices including smartphones and laptop computer.

**Table 2:** Ownership and experience with digital technologies (*N* = 45)

Variable	Pre-training (*n*, %)		Post-training (*n*, %)		*P*-value
	**Yes**	**No**	**Yes**	**No**	
**Ownership of Relevant Electronic Devices**					
Smartphone	41 (91.11)	4 (8.89)	43 (95.56)	2 (4.44)	0.625
Tablet	25 (55.56)	20 (44.44)	26 (57.78)	19 (42.22)	***0.016***
Laptop Computer	39 (86.67)	6 (13.33)	39 (86.67)	6 (13.33)	1.000
Desktop Computer	14 (31.11)	31 (68.89)	18 (40.00)	27 (60.00)	0.317
**Experience with Digital Technologies**					
Use Videos in Teaching	29 (64.44)	16 (35.56)	35 (77.78)	10 (22.22)	0.180
Participated in online programme	25 (55.56)	20 (44.44)	29 (64.44)	16 (35.56)	0.285
Use of Microsoft PowerPoint with voice recording in Teaching	29 (64.44)	16 (35.56)	34 (75.56)	11 (24.44)	0.197
Teach using Zoom	30 (66.67)	15 (33.33)	34 (75.56)	11 (24.44)	0.317
Experience with Google Meet	15 (33.33)	30 (66.67)	18 (40.00)	27 (60.00)	0.439
Experience with Google Class	14 (31.11)	31 (68.89)	22 (48.89)	23 (51.11)	*0.045*
Experience with online library resources	21 (46.67)	24 (53.33)	30 (66.67)	15 (33.33)	***0.039***

Table 3 presents the perceived usefulness of technology in clinical teaching; it shows a gain in perception of usefulness regarding items including “using technology in my job would enable me to accomplish objectives more quickly” as it increased from 40 (88.89%) to 43 (95.56%) after the intervention. Regarding the statement that “using technology will improve clinical teaching” there was a gain from 19 (42.22%) before the training intervention to 44 (97.78%) after the intervention. In connection with the perceived ease of using technology in clinical teaching, among those who agreed to the statement that “I would find it easy to get this technology to do what I want to do” there was an increase from 36 (80.00%) before the training to 41 (91.11%) after the training intervention. Similarly, among those who agreed to the statement that “It will be easy for me to become skillful in the use of this technology”, there was a gain after the intervention from 41 (91.11%) to 43 (95.56). Additionally, the participants’ intention to use technology increased from 42 (93.33%) to 44 (97.78%) and self-efficacy increased from 40 (88.89%) to 43 (95.56%) after the intervention, respectively.

**Table 3:** Perceived usefulness, ease of use, intention to use and self-efficacy among participants pre and post intervention (*N* = 45)

Variable	Pre-training (n, %)			Post-training (n, %)		
	**Agreement**	**Neutral**	**Disagreement**	**Agreement**	**Neutral**	**Disagreement**
**Perceived useful of technology in clinical teaching**						
Using technology in my job would enable me to accomplish objectives more quickly	40 (88.89)	3 (6.67)	2 (4.44)	43 (95.56)	1 (2.22)	1 (2.22)
Using the technology will improve clinical teaching	19 (42.22)	1 (2.22)	25 (55.56)	44 (97.78)	0 (0.00)	1 (2.22)
Using the Technology at work will improve productivity	43 (95.56)	1 (2.22)	1 (2.22)	43 (95.56)	0 (0.00)	2 (4.44)
Using the technology will enhance my effectiveness in my job	44 (97.78)	0 (0.00)	1 (2.22)	43 (95.56)	0 (0.00)	2 (4.44)
Using technology will make it easier to do my job	44 (97.78)	0 (0.00)	1 (2.22)	44 (97.78)	0 (0.00)	1 (2.22)
**Perceived ease of use of technology in clinical teaching**						
Learning to use technology in my work will be easy for me	44 (97.78)	0 (0.00)	1 (2.22)	44 (97.78)	0 (0.00)	1 (2.22)
I would find it easy to get this technology to do what I want it to do	36 (80.00)	4 (8.89)	5 (11.11)	41 (91.11)	3 (6.67)	1 (2.22)
It will be easy for me to become skillful in the use of this technology	41 (91.11)	3 (6.67)	1 (2.22)	43 (95.56)	0 (0.00)	2 (4.44)
**Intention to use and self-efficacy of preceptors toward technology in clinical teaching**						
I presently intend to use the technology regularly at work	42 (93.33)	2 (4.44)	1 (2.22)	44 (97.78)	0 (0.00)	1 (2.22)
I will find the technology easy to use	43 (95.56)	2 (4.44)	0 (0.00)	44 (97.78)	0 (0.00)	1 (2.22)
I feel confident in using technology in clinical teaching	40 (88.89)	5 (11.11)	0 (0.00)	43 (95.56)	0 (0.00)	2 (4.44)

For the qualitative dataset, Table 4 shows that seven nurse preceptors participated which included five females and two males, with 10 to 20 years of experience as clinicians. They have been involved in clinical teaching for a minimum of two years and maximum of eleven years. Participant’s age ranged from 35 to 55 years.

**Table 4:** Sociodemographic characteristics of the participants

Participant	Age	Gender	Work Experience	Rank	Clinical Specialty	Years of Precepting	Educational Background	Type of Clinical Site
1	35	Male	13	Senior Nursing Officer	Adult Health	9	B.Sc. Nursing	Teaching Hospital
2	37	Male	12	Senior Nursing Officer	Adult Health	1	M.Sc. Nursing	District
3	42	Female	19	Principal Nursing Officer	ENT	10	M.Sc. Nursing	Teaching Hospital
4	55	Female	20	Principal Nursing Officer	Adult Health	7	B.Sc. Nursing	Teaching Hospital
5	41	Female	19	Deputy Director of Nursing Services	Critical thinking	11	Master of Nursing	Teaching Hospital
6	40	Female	18	Principal Nursing Officer	Emergency Nursing	10	Master of Nursing	Teaching Hospital
7	36	Female	15	Principal Nursing Officer	Public Health	2	MPH	Hospital

They employed zoom and WhatsApp in engaging students using tablets, phones, and laptops as the main devices. The results yielded three themes, namely: strengths of using technology; constraints in the learning environment; and future of technological approach to clinical teaching.

## Theme I: Strengths of using technology

This theme highlighted participants’ general perception of using technology in clinical teaching. The participants perceived the use of technology in clinical teaching to be in tandem with “*modern era”* (P1) and helpful amidst the COVID-19 pandemic (P5). Two subthemes emerged out of this theme, namely: positive perception, and conducive atmosphere for improved learning outcomes.

### Subtheme A: positive perception

Participants were receptive to the idea of using technology in clinical teaching. They felt that it was “*nice*” (P1, P2), “*awesome”* (P2), “*good…, timely*” (P4), “*interesting*” (P5) and “*very useful*” (P6) approach to clinical teaching. Also, they thought the use of technology in clinical teaching was “*better than face-to-face*” approach (P2), “easier” to face-to-face approach (P3), “*helps both students and preceptors*” (P1), and “*boosted [ preceptors’] morale”* (P2). A participant (P5) thought “*in-person [face-to-face] interaction was overrated”)*. Yet, the usefulness of technology in clinical teaching was dependent on good access to technology itself (P5).

### Subtheme B: Conducive atmosphere for improved learning outcomes

Participants were of the view that technology could potentially provide conducive atmosphere to improved clinical learning outcomes. Participants highlighted the fact that a lot more students could be reached with technology as epitomized by the following quote:

*“I think we can be able to reach more and more students and then get more time compared to the physical [face-to-face approach]”* (P1).

Participants also felt the use of technology in clinical teaching encouraged preceptors to source for information for students: *“You can explore a lot of other resources from online*” (P2) and convenient in many ways: “*you can always do it*” (P6); *we can…reach out to the students anytime, anywhere that we find ourselves* (P1). Additionally, participants were innovative toward students’ learning. A participant (P5) shared her excitement making a video on a procedure for students:

“*I also like the part where could make videos and share with them [students]…how can I create content for these students”* (P5).

Participants further thought students learnt better because workplace interruption and workload of preceptors that may be the hallmark of face-to-face clinical sessions were removed:

*“Teaching…without…struggle with…interruptions that may come from other people wanting to work on the patient”* (P1).

“*With the use of the technology, at least you, the interference at work is minimized* (P6).

Furthermore, the use of technology such as videos provided the avenue for students to go over lessons that enhanced learning outcomes:

“*Student will have the opportunity to watch over again, watch over again, watch over again”* (P2:)

“*The feedback shows that they were getting the things right*” (P4).

Similarly, participants believed the use of technology encouraged healthy relationship between them and students which is epitomized by the following quote:

“*We've built a relationship with our students. They can call…at any time if they need something. If they want to discuss something with me, they are free”* (P4)

## Theme II: Constraints in the learning environment

Participants identified factors in the learning environment that could limit the use of technology in clinical teaching. The constraints in the learning environments included six subthemes, namely: infrastructure constraints, inherent constraint of technological approach, student factors, preceptor factors, institutional factors, and time constraints.

### Subtheme A: Infrastructure constraints

Participants identified some impairment that inhibited the use of technology in clinical teaching. Absence or poor internet connectivity was seen as crucial impairment: *“they always complained about the internet*” (P1), “*our major problem was network*” (P2). Also, lack of devices was identified as a barrier: “*if you don't have the device that [you] would be able to use…then it becomes a problem*” (P5).

Another challenge was unstable power supply: “*Sometimes you'll be teaching…sudden[ly] the light will go off, that one is a challenge*” (P4).

### Subtheme B: Inherent constraint of technological approach

There were some inherent challenges participants identified that could limit the use of technology in clinical teaching. One of such challenge is its practicality in certain learning moments such as evaluating of students’ learning outcome as demonstrated by the following quotes:

“*It's a bit difficult to really evaluate whether the students are getting what you are telling them. This is a typical example, when I told them, “Apply the cuff",…"Wear your stethoscope", I could see…. But I couldn't evaluate what they were hearing*” (P5).

*“I had wanted to demonstrate certain things to them. But because it was online…I didn't know how to do the demonstration*” (P4).

### Subtheme C: Student factors

Participants identified student factors that could limit the use of technology in clinical teaching. Some of these factors included **s**tudents’ attendance or attention: “*They will log in and… they will not pay attention…The students were not forthcoming, you call, you schedule a time to come and [they will] give you stories,”* (P6).

Some students were constrained financially: *“some of the students were not…getting money to buy [internet] data*” (P4).

Lack of seriousness or commitment was identified by participants to hinder learning with the use *of technology:*

### Subtheme D: Preceptor factors

The preceptor factors as a subtheme, were preceptor-related constraints to the use of technology. Constraints relating to the busy schedules of preceptors were cited by participants as a potential hindrance to the use technology in clinical teaching:

*“Sometimes, you will fix a time, … You come and the ward is too heavy and… you…have to tell the student to… rescheduled the time, and…it disturbs them” (*P2).

Similarly, unfamiliarity of use of technology was identified as a preceptor-related barrier: “*that was my first time of using zoom to teach somebody. I had not used zoom before…I was having challenges when it had to be used to teach students*” (P2).

### Subtheme E: institutional factors

Some institutional factors were identified as barriers to the use of technology in clinical teaching.

One participant cited ineffective collaboration as one of the barriers: “*on paper, collaboration exist but in reality, it doesn't. Because there is a disjoint between the faculty and the preceptors*” (P6).

Another identified ineffective communication as a barrier: “*I don't know the kind of communication that went [on]… they had other lectures with certain faculty members and they saw that one as more important than the one they had*” (P1).

### Subtheme F: time constraints

Scheduling of sessions sometimes coincided with other activities: “*we schedule our days together, but getting to… the period, they were complaining, they were having exams, they were having other sessions with other lecturers. So, it was interfering with our meeting”* (P3).

## Theme III: Future of technological approach to clinical teaching

This theme relates to participants’ views on the prospects of the use of technology in clinical teaching. All participants thought that the future of clinical teaching is routed in technology. This view was epitomized by these assertions:

“*Technology, forward ever and backward never*” (P5). “*it's a good exercise. In this modern era, we need to continue*” (P1).

Participants identified two factors (subthemes) that are required to make the future use of technology in clinical teaching plausible: enablers of the use of technology and institutional alignment and support.

### Subtheme A: enablers of the use of technology

Participants asserted that to depend on technology in clinical teaching, some enabling factors should be put in place. These enabling factors should be directed at supporting students, including the training of students on online resources, provision of internet data bundle:

“*Then if they can be supported with internet [data]”* (P7).

*“[The school should] arrange with the networking company, so that they will give them a special package”* (P2).

*“[The school) should teach the student how to explore online [resources]. So that they will not have problem[s]”* (P2).

Participants also noted that to use technology effectively, preceptors should be supported:

“*The school [should] train the instructors on the need to get themselves acquainted with a lot of online platforms as to how to use to teach students*” (P2).

### Subtheme B: institutional alignment and support

Another area participants felt could ensure the future use of technology in clinical teaching was institutional alignment and support to accommodate technology. Participants thought using contextually made videos were avenues nursing schools should align themselves:

“*You know some of the videos that I used … were not within our Ghanaian setting…if [we] are able to… record it [videos] I think that one can also help us”* (P1).

Blended approach to clinical teaching was seen by participants as the ultimate approach:

*“Blended approach…I think it's the best I mean*” (P2).

Participants asserted that using collaborative assessment strategy will get students to be committed to the use of technology in clinical teaching:

*“Students…feel that we don't have any inputs in their grades. And you know studentsvalue grades a lot, because they value grades because of grades that's why somebody willcome for clinical session”* (P7).

## Discussion

### Ownership and experience with the use of digital technologies

Our study sought to investigate nurse preceptors’ ownership and experience with the use of digital technologies, perceptions, intention to use and self-efficacy towards digital technology in preceptorship and challenges to the use of technology in preceptorship using mixed-methods approach. The use of digital technologies in clinical teaching is critical, especially in this era of COVID-19 pandemic. Our findings indicated that apart from tablets, there was no statistically significant increase in the ownership of relevant electronic devices by nurse preceptors after the training intervention (*p*-value of  > 0.05). Even though, ownership of smartphones and desktop computers increased, the results were not statistically significant (*p*-value of (0.625 and 0.317 respectively). This can be due to the reason that participants acquired the knowledge during training that our everyday handheld devices could be used to introduce technology into our teaching at the clinical site [19, 20]. Also, participants’ experiences with other forms of digital technology did no increase significantly except that of online library resources (*p* = 0.039).

### Perceptions of digital technology in preceptorship

In our study, the participants perceived technology to be useful in their clinical teaching, as evidenced by improvement in perceived usefulness regarding the statement “using technology in my job would enable me to accomplish objectives more quickly”, which increased from 88.89% at baseline to 95.56% post training intervention. Additionally, the participants agreed to the statement that “using technology will improve clinical teaching”, as there was a gain from 42.22% before the training intervention, to 97.78% after the training intervention. Perceived usefulness is the degree to which an individual acknowledges the importance that technology can enhance performance of an activity [21]. Therefore, if one believes that technology can facilitate clinical teaching one might use it during the teaching process.

It is not surprising that in the post intervention qualitative study, the participants expressed a positive perception of the use of technology in clinical teaching. They viewed technology as able to potentially provide a conducive environment for improved learning outcomes, encourage nurse preceptors to source for information for students, and reduce the interruptions students experience during face-to-face clinical teaching sessions. This support literature citing that technology in clinical education enables the facilitator to access reference materials, for both faculty and students, and educators had a positive attitude toward its use [22, 23]. A plausible explanation could be that the participants sampled had an improved appreciation about the immense benefits of technology in nursing education, following the training/intervention. Innovations and transformations in nursing education, including the incorporation of twenty-first century skills, have necessitated efforts for educators to shape the learning environment by embracing technology in delivering quality nursing education. The use of digital technology is essential in transforming the twenty-first century learning environment [24].

Furthermore, this study investigated perceived ease of use of technology. Perceived ease of use is the assumption that using technology will not require so much effort. This is somewhat influenced by the individual skills and competencies needed to man the system [25]. It is an important construct underpinning attitudes towards the perceived ease of use of technology. In the current study, participants’ perception that it would be easier to get technology to do what they wanted to do, and also become skillful in the use of technology, increased after the intervention. Therefore, the intervention might have influenced the perceptions of the participants regarding ease of use of technology. An earlier study found nurses to be more optimistic and innovative regarding their readiness to use technology but were uncomfortable about its use [26].

### Challenges to the use of technology in preceptorship

Despite a gain in perceived ease of use regarding accessibility and competence in using technology post intervention, the interviewees highlighted some factors which hindered the use of technology in clinical teaching. As in other parts of the world, there have been efforts by educators to incorporate technology into their array of teaching methods; however, there are challenges associated with its use, and appropriately using them to their fullest capability [27]. Some of the challenges identified in our study, were associated with the clinical environment (poor internet connectivity and unstable power supply), constraint of technological approach (practicality in certain learning moments), student factors (lack of seriousness and commitment), preceptor factors (heavy work load, unfamiliarity of use of technology and time constraints), and institutional factors (ineffective collaboration and communication between faculty and preceptors). These barriers have also been identified in other studies [20, 23, 28–31]. For instance, in a study conducted among international medical students in China, poor internet connectivity was cited as a major challenge the students and some faculty encountered during their online sessions [32] Moreso, studies in high-income economies have reported similar challenges regarding students online learning [31, 33]

### Intention to use and self-efficacy regarding technology in clinical preceptorship

There were gains in participants’ intention to use and self-efficacy regarding technology in clinical teaching. They believed it will be easy to do, and felt confident to incorporate its use in their teaching. The participants’ perceived ease of use and perceived usefulness of technology may have influenced their intention to use technology in clinical teaching [34, 35]. This was supported by the narratives of the qualitative interviews conducted post intervention. There were prospects of the use of technology in clinical teaching in the future. All participants thought that the future of clinical teaching is routed in technology and that some enabling factors should be put in place to achieve this. Institutional alignment, in the form of using videos with local content for teaching, as well as the need to accommodate the use of technology for clinical teaching by supporting institutions, was emphasized.

## Conclusions

Clinical education gives students the opportunity to learn in real-life conditions, applying theory to practice. This makes it an inseparable part of nursing education. Effective clinical nursing education depends on preceptors to facilitate the transition of the preceptee, from student to professional nurse. This training intervention improved participants intentions, self-efficacy, perceived use and perceived ease of use of technology. However, there were constraints in the clinical learning environment, including students and preceptor-related factors and institutional factors that need to be addressed as part of efforts to efficiently implement technology in clinical teaching. The findings have implications for shaping clinical nursing education in this era of COVID-19 pandemic and beyond.

A major strength of this study is the use of the multimethod approach to capture diverse information about the phenomenon. A clear limitation worth mentioning is the small sample size, and this therefore influences the recommendations from the study. Further studies could explore interventions that support virtual teaching in the clinical environment in low-to-middle-income economies using multiple sites with larger number of preceptors.

## Data Availability

The datasets used and/or analysed during the current study are available from the corresponding author on reasonable request.

## Abbreviation

COVID 19: Coronavirus disease 2019
